# Frequency of Epidermal Growth Factor Receptor and T790M Mutations Among Patients With Non-Small Cell Lung Carcinoma: A Hospital-Based Study in the King Khalid University Hospital (KKUH) Since 2009-2017

**DOI:** 10.7759/cureus.19816

**Published:** 2021-11-22

**Authors:** Sara H AlQahtani, Areeb M AlOgaiel, Kowthar N AlMosa, Suha H Alenazi, Monira K AlHasan, Reham H AlObaidan, Bayan D Aldokheel, Khalid AlSaleh, Maha Arafah, Imran Ali Khan, Maram AlOtaiby

**Affiliations:** 1 Medicine, King Saud University, Riyadh, SAU; 2 General Surgery, King Abdullah Bin Abdulaziz University Hospital, Riyadh, SAU; 3 Medicine, University of Hail College of Medicine, Hail, SAU; 4 College of Medicine, King Saud University, Riyadh, SAU; 5 Pathology, King Saud University, Riyadh, SAU; 6 Clinical Laboratory Sciences, College of Applied Medical Sciences, King Saud University, Riyadh, SAU; 7 Molecular Genetics Pathology Unit, Department of Pathology, King Saud University, Riyadh, SAU

**Keywords:** egfr, non-small cell lung carcinoma (nsclc), t790m, frequency, saudi arabia

## Abstract

Objectives

To estimate the proportion of positive epidermal growth factor receptor (EGFR) mutations among patients diagnosed with non-small cell lung carcinoma (NSCLC) and T790M at the King Khalid University Hospital (KKUH).

Methods

A retrospective cohort study that included all patients that were diagnosed with NSCLC from 2009 to 2017 at KKUH. Data obtained from both electronic and paper medical records and the following information were studied: age, gender, smoking, region, subtype of NSCLC, EGFR mutation test result, treatment, T790M mutation test (if required), comorbidities, metastasis. Statistical analysis was performed using the Statistical Package for the Social Sciences (SPSS, version 21.0; SPSS Inc., Chicago, IL, USA).

Results

Among 71 patients with NSCLC 18 cases were identified for EGFR positive mutation and only one case for T790M. Deletion mutation in exon 19 represented 50% of total cases. Moreover, it showed that it is more frequent in males and non-smokers with 61.1% (11) and 66.7% (12), respectively. Majority of the cases were above the age of 60 years by 61.1% (11). The mutations reported highest in those living in Najd with a 44.4% (8) and all the mutated cases were adenocarcinoma. There was no statistical significance in the association between EGFR mutation and disease variables.

Conclusion

Ultimately, we found that the frequency of EGFR and T790M mutations among NSCLC patients at KKUH from 2009 to 2017 was 25.4% and 1.4%, respectively. Moreover, this result was conspicuous among non-smokers.

## Introduction

Lung cancer is the second most common cancer among both men and women in the United States and it represents 5% of all cancers in Saudi Arabia [1]. Non-small cell lung cancer (NSCLC), accounts for 85% of all lung cancer cases and it is the most common primary malignancy leading to synchronous brain metastases [2]. Like other cancers, it is initiated by activating oncogenes or inactivating tumor suppressor genes, which mainly occur through epigenetic changes [3]. It is widely known that smoking is strongly linked to the increased incidence of lung cancer, another risk factor lies in the genes; a mutation of epidermal growth factor receptor (EGFR) or TP53 was reported in patients with NSCLC [4]. Subtypes of NSCLC were grouped into one category due to the insignificance of histological classification on the treatment plan. On the other hand, recent studies showed the impact of molecular differentiation on positive EGFR adenocarcinoma with targeted therapy, as it became essential for future therapeutic plans [5]. 

EGFR is a transmembrane receptor with tyrosine kinase activity that is responsible for facilitating many intracellular signaling pathways [6-8]. The EGFR gene is located on the short arm of chromosome number 7 at position 11.2 [9-10]. If the EGFR gene is mutated it will lead to an increase in cell division, angiogenesis, migration, and metastasis; which are the cellular characteristics of cancer [11]. Deletions and point mutation in exon 19 and 21, respectively are found in 85% of the cases [12]. Many studies showed that EGFR mutations are associated with adenocarcinoma, non-smokers, females, and Asians [8,13]. A meta-analysis study reported that the worldwide prevalence of EGFR mutation is 32.3%. The highest number is reported in China 38.4%, followed by Uruguayan population 18.3%, Europe 14.1%, and Caucasian 5.4% [14-16]. Regionally, a study in Levant area including Jordan, Syria, Lebanon reported 15.6% [17], and 20% in Morocco [18]. Two studies have been conducted in Saudi Arabia, one of them was not suitable for statistical analysis due to the low number of cases [19]. The second study included the Gulf countries and reported the prevalence of EGFR mutations in 28.7%. However, it was not specific for the Saudi population. [20]

The presence of the EGFR gene mutations alters the prognosis, as EGFR mutations are found to be highly sensitive to tyrosine kinase inhibitors (TKIs) [21]. Response rates to TKIs in all cases were 50% to 100% in patients with mutant EGFR tumors [22]. As suggested by recent trials and guidelines, TKIs as an initial therapy is associated with an overall better survival rate, enhancement in the quality of life and reduction in therapy-related side effects compared to patients who have been treated with chemotherapy [14,21-22]. The efficiency of the TKI treatment is limited by the development of a mutation in the T790M gene resulting in an acquired resistance [21]. A study was conducted in Japan, found that 48% of patients developed T790M mutation after they have been treated with TKIs. Patients who acquired T790M after the failure of EGFR-TKIs had better response rates and progression-free survival on initial EGFR-TKI than those without T790M. A newer generation of TKIs is currently used for patients who acquired T790M mutation [13].

T790M mutation is an acquired drug-resistant mutation that developed after treating NSCLC patients having EGFR mutation with TKIs. It is a secondary point mutation that substitutes methionine for threonine at amino acid position 790 of exon 20 [23]. Multiple studies noticed that gender, initial EGFR-TKI response, and progression patterns were significantly associated with T790M mutation status, while others showed no relation to gender, smoking or histology but found that the response is more frequent in advanced-stage tumors than in early-stage tumors [24-26]. In a study of 99 patients with EGFR mutation, a re-biopsy was done to detect T790M mutation and reported an overall prevalence of 68%.

Due to the current scarcity of studies regarding EGFR and T790M mutations in Saudi Arabia and limitations in previous studies such as a low number of cases, the implementation of first-line treatment is not generalized in our hospitals and yet replaced by chemotherapy. Therefore, testing for the mutation in the EGFR gene and understanding its prevalence is mandatory to be identified in the patients with NSCLC as it will alter the prognosis, nature of the treatment, the pattern of progression and the overall outcome [22,27]. We aim in this study to estimate the frequency of positive EGFR and T790M mutations among NSCLC patients at king Khalid university hospital and to identify any association with the clinical or demographical characteristics. Thus, the aim of this study is to estimate the proportion of positive EGFR mutations among patients diagnosed with non-small cell lung carcinoma (NSCLC) and T790M at King Khalid University Hospital (KKUH).

## Materials and methods

This is an observational quantitative retrospective cohort study that was held at KKUH, Riyadh, Saudi Arabia from January 1st, until May 15th, 2018. On NSCLC patients of both genders from 2009 to 2017. The study included all patients with NSCLC with a total number of 150 patients. However, due to fragmented and inadequate documentation, only 71 subjects were included in this study. In this study, all patients were diagnosed with NSCLC and have done EGFR and T790M (if required) mutation test from 2009 to 2017 at KKUH. All files’ numbers of NSCLC patients were requested from the Oncology unit, then patients’ pathological reports from the pathology department were obtained to extract mutation tests results, also clinical and demographical data relevant to our study obtained from both electronic and paper medical records. All data were recorded in a transfer sheet. 

The following information were extracted: demographic data: (age, gender, smoking, region) and clinical data: (subtype of NSCLC, stage/grade, EGFR mutation test result, treatment, T790M mutation test result if resistant to TKIs and co-morbidities).

The inclusion criteria were as follows: NSCLC (inpatient and outpatient), have done EGFR and T790M (if required) mutation test and complete medical record. The exclusion criteria were positive EGFR mutation who received chemotherapy as the first-line of treatment and files with incomplete data. 

Data analysis

Statistical analysis was performed using the Statistical Package for the Social Sciences (SPSS) Pc + 21.0 version statistical software (SPSS Inc., Chicago, IL, USA). Descriptive statistics (frequencies, percentages, mean and standard deviation) were used to describe the categorical and quantitative variables. In addition, a univariate and multivariate analysis was performed to determine the associations between positive EGFR cases with demographical and clinical characteristics. A p-value of ≤ 0.05 was considered significant and 95% confidence intervals were used to report the statistical significance and precision of results.

Ethical considerations

An ethical approval from the Institutional Review Board at King Saud University prior to the start of the study was obtained from the Institutional Review Board at the College of Medicine at King Saud University (Project No. CMED 305-F13-2017-18). Participants’ information encrypted (patient ID) and confidentiality is granted. Informed consent is not required in this study, as the data is obtained from medical records.

## Results

Among 71 patients with NSCLC, the mean age was 60 years with a standard deviation of 13.83. Smoking status, the region of the included patients, presence of comorbidities and subtypes of NSCLC are shown in Table 1. 

**Table 1:** Socio-demographics and clinical characteristics among NSCLC patients (n = 71). NSCLC: non-small cell lung carcinoma.

Variables	Frequencies (%)
Age groups	
< 60 years	34 (47.9)
≥ 60 years	37 (52.1)
Gender	
Male	47 (66.2)
Female	24 (33.8)
Smoking	
Smoker	34 (47.9)
Non-smoker	37 (52.1)
Region	
Central Region (Najd)	42 (59.2)
Southern Region	5 (7)
Northern Region	5 (7)
Out of KSA	19 (26.8)
Comorbidities	
Diabetes mellitus	24 (33.8)
Hypertension	28 (39.4)
Dyslipidemia	4 (5.6)
Ischemic heart diseases	8 (11.3)
Benign prostatic hyperplasia (n = 47)	5 (10.6)
Others	18 (25.4)
Subtype of NSCLC	
Adenocarcinoma	64 (90.1)
Squamous cell carcinoma	7 (9.9)
Variable	Mean (SD)
Age	60.24 (13.83)

The EGFR mutations were present in 25.4% (18) patients. Further details about the characteristics of the mutations were obtained. These mutations occur within EGFR exons 18-21. However, there were no cases with exon 18, while half 50% of the patients had mutation in the exon 19 (deletion). Subtypes, presence of metastasis and other characteristics are shown in Table 2. 

**Table 2:** Non-small cell lung cancer mutations. EGFR: epidermal growth factor receptor.

Variables	Frequencies (%)
EGFR mutation (n = 71)	
Positive	18 (25.4)
Negative	53 (74.6)
Distribution of characteristics in positive EGFR mutation patients (n = 18)	
Types	
Exon 18	0
Exon 19 (deletion)	9 (50)
Exon 20 (insertion)	3 (16.7)
Exon 21 (insertion)	6 (33.3)
Age	
< 60 years	7 (38.9)
≥ 60 years	11 (61.1)
Gender	
Male	11 (61.1)
Female	7 (38.9)
Smoking	
Smoker	6 (33.3)
Non-smoker	12 (66.7)
Region	
Central Region (Najd)	8 (44.4)
Southern Region	3 (16.7)
Out of KSA	7 (38.9)
Subtype	
Adenocarcinoma	18 (100)
Metastasis	
Positive	16 (88.9)
Negative	2 (11.1)
Comorbidities	
Diabetes mellitus	9 (50)
Hypertension	10 (55.6)
Ischemic heart diseases	2 (11.1)
Benign prostatic hyperplasia (n = 11)	2 (18.8)
T790M mutation (n = 18)	
Positive	1 (5.6)
Negative	3 (16.7)
Not performed	14 (77.8)

There was no statistical significance in the association between EGFR mutation and the following variables: age, gender, smoking, region, subtype, metastasis, diabetes mellitus, hypertension, dyslipidemia, ischemic heart disease, and benign prostatic hyperplasia (Table 3). 

**Table 3:** Association of EGFR mutation with socio-demographics and clinical characteristics. EGFR: epidermal growth factor receptor.

Variables	No. of positive cases n (%)	Odds ratio	95% confidence interval	p-value
Age				
< 60 years	7 (38.9)	0.613	0.206-1.823	0.376
≥ 60 years	11 (61.1)			
Gender				
Male	11 (61.1)	1.34	0.444-4.086	0.598
Female	7 (38.9)			
Smoking				
Smoker	6 (33.3)	2.240	0.732-6.856	0.153
Non-smoker	12 (66.7)			
Region				
Central Region (Najd)	8 (44.4)	2.479	0.740-8.307	0.141
Southern Region	3 (16.7)	0.389	0.052-2.924	0.359
Out of KSA	7 (38.9)	0.46	0.146-1.446	0.184
Subtype				
Adenocarcinoma	18 (100)	0.868	0.781-0.964	0.179
Metastasis				
Positive	16 (88.9)	0.611	0.119-3.136	0.718
Negative	2 (11.1)			
Comorbidities				
Diabetes mellitus	9 (50)	2.533	0.843-7.613	0.093
Hypertension	10 (55.6)	2.431	0.817-7.227	0.105
Ischemic heart diseases	2 (11.1)	0.979	0.179-5.349	0.981
Benign prostatic hyperplasia	2 (18.8)	0.480	0.074-3.132	0.595

Different modalities for treatment among patients were also reported. Chemotherapy was used for the majority of patients 95.9%. Among the EGFR mutated patients (n = 18), one or more modalities have been used; chemotherapy in 88.9% (16) and TKI in 66.6% (12). While 11.1% (2) of EGFR mutated cases were treated surgically, and 16.7% (3) of them underwent radiotherapy. Lastly, only one patient developed the T790M gene mutation and was managed by different TKIs (Erlotinib, Osimertinib and Afatinib) in addition to radiotherapy (Table 4).

**Table 4:** Management options for non-small cell lung cancer patients.

Variables	Frequencies (%)
	Total (n = 71)
Chemotherapy	68 (95.9)
Carboplatin	19 (26.8)
Pemetrexed	18 (25.4)
Cisplatin	9 (12.7)
Gemcitabine	8 (11.3)
Oxaliplatin	1 (1.4)
Docetaxel anhydrous	2 (2.8)
Others	11 (15.5)
Tyrosine kinase inhibitors	27 (38)
Erlotinib	21 (29.6)
Osimertinib	2 (2.8)
Crizotinib	3 (4.2)
Afatinib	1 (1.4)
Others	22 (31)
Surgery	7 (9.9)
Radiotherapy	15 (21.1)

## Discussion

The significance of this study sheds the light on establishing the frequency of EGFR and T790M mutations among our targeted population. It was found that the frequency of EGFR mutated NSCLC patients is 25.4% of all cases, with the highest number in Najd (central region), followed by non-Saudis and the least number was found to be in the southern region, while no other available researches in Saudi Arabia are present at the moment to compare with. Nevertheless, in the Middle Eastern region the only study of lung cancer-specific of EGFR mutation to date involves the levant population, the EGFR mutation was founded in 15.6% of NSCLC patients, which is lower than ours [17]. Moreover, the international numbers revealed a great variation; a higher number found to be in China 38.4%, followed by the Uruguayan population 18.3%, Europe is 14.1%, and the Caucasians 5.4% [14-16]. On the scale of Saudi Arabia, Levant region and internationally (China and Caucasian population), adenocarcinoma was found to be the subtype associated with the highest numbers of EGFR mutations. In addition, the mean age of our chosen sample at which the mutation was detected is 60.2 years, which was consistent with the mean age of the levant population 63.4 years, but unfortunately this variable was not taken into consideration by the international studies. As stated by our gender distribution, male gender prevalence is higher than females, which aligns greatly with the levant population and the Uruguayan population. Diversely, the Caucasians showed no difference in developing the mutation regarding gender distribution. In terms of smoking history, 66.7% of non-smokers had the mutation, on the other hand, only 33.3% were smokers. Those findings are consistent with the ones found in the Levant and the Chinese population. A study in the United States showed that higher mutation rates lie within patients who never smoke, thus they are more sensitive to TKIs [28], this demonstrates the importance of early mutation detection and intervention as it carries better prognosis chances for such patients. Furthermore, the presence of the EGFR mutation and distant metastasis (stage IV) was higher in comparison with the presence of the mutation in lesser advanced stages. Those findings were supported by the state of our patients, Levant region and international (China and Caucasian) patients. With respect to the existence of comorbidities, hypertension happened to be the highest comorbidity among EGFR mutated patients in our and the Levant population. On the contrary, no international sufficient data in regard to the comorbidities. 

Regarding the T790M mutation, only one patient developed this mutation among our population. Similarly, the power of the T790M results does not support giving conclusions while based on one patient only. Strangely enough, two separate studies in Japan reported almost half of the population developed this mutation [24]. The differences in the results might be due to environmental, genetical, geographical factors, gender distribution, the initial response to the EGFR-TKI treatment, and the progression pattern of the cancer. Hopefully, future researches might aid in showing the reasons behind those variations. 

Understanding the variety of the recommended management plans that target the EGFR and T790M mutations is another major aspect of this study. Upon our population, four different management plans were considered, shockingly and although evidence-based medicine guidelines recommended TKIs as a first-line therapy for EGFR mutated NSCLC, they came second to chemotherapy in terms of the most commonly used treatment. Consequently, followed by surgery and then radiotherapy. Meanwhile, nearly matching order of management plans was applied on the levant population, except the absence of the TKIs usage. In addition to that, radiotherapy is being used more frequently than surgery. Contrary-wise, based on a meta-analysis, the non-chemotherapy approach was more popular.

In spite of the fact that the research has reached its aims, there have been some inevitable limitations. First, the highest prevalence was in the central region, most probably due to conducting our research at KKUH. Second, incomplete secondary data as a result of fragmented and inadequate documentation. Third, owing to the limited timeframe, we were not able to execute it as a multi-center research. Fourth, a small sample size that might led to overestimated values and constrained the ability to generalize the findings, in view of the reasons mentioned above. Finally, the total absence of facilities/laboratories to detect the targeted mutations in Saudi Arabia drove us to the use of a retrospective cohort as the chosen study design. 

## Conclusions

Ultimately, we found that the frequency of EGFR and T790M mutations among NSCLC patients at KKUH from 2009 to 2017 was 25.4% and 1.4%, respectively. Moreover, this result was conspicuous among non-smokers. A delay in starting the appropriate therapy has been observed owing to dispatching the samples overseas to get them tested for the mutations despite the high prevalence (25.4%) of EGFR mutated cases among our population, hence we recommend the implementation of EGFR mutation test in Saudi Arabia. We also suggest developing methods and tools for earlier detection as we noticed that most of the positive EGFR mutations were detected in stage IV of the disease which is indicative of bad prognosis. Together with the previous recommendations, we stand by establishing a national registry for lung cancer and its mutations, as well as allowing accessibility for researchers.

## References

[1] Dela Cruz CS, Tanoue LT, Matthay RA (2011). Lung cancer: epidemiology, etiology, and prevention. Clin Chest Med.

[2] Herbst RS, Heymach JV, Lippman SM (2008). Lung cancer. N Engl J Med.

[3] Johnson L, Mercer K, Greenbaum D (2001). Somatic activation of the K-ras oncogene causes early onset lung cancer in mice. Nature.

[4] Molina JR, Yang P, Cassivi SD, Schild SE, Adjei AA (2008). Non-small cell lung cancer: epidemiology, risk factors, treatment, and survivorship. Mayo Clin Proc.

[5] Al Dayel F (2012). EGFR mutation testing in non-small cell lung cancer (NSCLC). J Infect Public Health.

[6] Lohinai Z, Hoda MA, Fabian K (2015). Distinct epidemiology and clinical consequence of classic versus rare EGFR mutations in lung adenocarcinoma. J Thorac Oncol.

[7] Lindeman NI, Cagle PT, Beasley MB (2013). Molecular testing guideline for selection of lung cancer patients for EGFR and ALK tyrosine kinase inhibitors: guideline from the College of American Pathologists, International Association for the Study of Lung Cancer, and Association for Molecular Pathology. J Thorac Oncol.

[8] Shigematsu H, Lin L, Takahashi T (2005). Clinical and biological features associated with epidermal growth factor receptor gene mutations in lung cancers. J Natl Cancer Inst.

[9] Francke U, Yang-Feng TL, Brissenden JE, Ullrich A (1986). Chromosomal mapping of genes involved in growth control. Cold Spring Harb Symp Quant Biol.

[10] Eley GD, Reiter JL, Pandita A, Park S, Jenkins RB, Maihle NJ, James CD (2002). A chromosomal region 7p11.2 transcript map: its development and application to the study of EGFR amplicons in glioblastoma. Neuro Oncol.

[11] Kosaka T, Yatabe Y, Endoh H, Kuwano H, Takahashi T, Mitsudomi T (2004). Mutations of the epidermal growth factor receptor gene in lung cancer: biological and clinical implications. Cancer Res.

[12] Yeh P, Chen H, Andrews J, Naser R, Pao W, Horn L (2013). DNA-Mutation Inventory to Refine and Enhance Cancer Treatment (DIRECT): a catalog of clinically relevant cancer mutations to enable genome-directed anticancer therapy. Clin Cancer Res.

[13] Jazieh AR, Al Kattan K, Bamousa A (2017). Saudi lung cancer management guidelines 2017. Ann Thorac Med.

[14] Zhang YL, Yuan JQ, Wang KF (2016). The prevalence of EGFR mutation in patients with non-small cell lung cancer: a systematic review and meta-analysis. Oncotarget.

[15] Berois N, Touya D, Ubillos L, Bertoni B, Osinaga E, Varangot M (2017). Prevalence of EGFR mutations in lung cancer in Uruguayan population. J Cancer Epidemiol.

[16] Skov BG, Høgdall E, Clementsen P (2015). The prevalence of EGFR mutations in non-small cell lung cancer in an unselected Caucasian population. APMIS.

[17] Tfayli A, Rafei H, Mina A (2017). Prevalence of EGFR and ALK Mutations in Lung Adenocarcinomas in the Levant Area - a Prospective Analysis. Asian Pac J Cancer Prev.

[18] Errihani H, Inrhaoun H, Boukir A (2013). Frequency and type of epidermal growth factor receptor mutations in moroccan patients with lung adenocarcinoma. J Thorac Oncol.

[19] Al-Kuraya K, Siraj AK, Bavi P (2006). High epidermal growth factor receptor amplification rate but low mutation frequency in Middle East lung cancer population. Hum Pathol.

[20] Jazieh AR, Jaafar H, Jaloudi M (2015). Patterns of epidermal growth factor receptor mutation in non-small-cell lung cancers in the Gulf region. Mol Clin Oncol.

[21] Kim JS, Cho MS, Nam JH, Kim HJ, Choi KW, Ryu JS (2017). Prognostic impact of EGFR mutation in non-small-cell lung cancer patients with family history of lung cancer. PLoS One.

[22] da Cunha Santos G, Shepherd FA, Tsao MS (2011). EGFR mutations and lung cancer. Annu Rev Pathol.

[23] Suda K, Onozato R, Yatabe Y, Mitsudomi T (2009). EGFR T790M mutation: a double role in lung cancer cell survival?. J Thorac Oncol.

[24] Oya Y, Yoshida T, Kuroda H (2017). Association between EGFR T790M status and progression patterns during initial EGFR-TKI treatment in patients harboring EGFR mutation. Clin Lung Cancer.

[25] Wang S, Cang S, Liu D (2016). Third-generation inhibitors targeting EGFR T790M mutation in advanced non-small cell lung cancer. J Hematol Oncol.

[26] Inukai M, Toyooka S, Ito S (2006). Presence of epidermal growth factor receptor gene T790M mutation as a minor clone in non-small cell lung cancer. Cancer Res.

[27] Rosell R, Moran T, Queralt C (2009). Screening for epidermal growth factor receptor mutations in lung cancer. N Engl J Med.

[28] Pao W, Miller V, Zakowski M (2004). EGF receptor gene mutations are common in lung cancers from "never smokers" and are associated with sensitivity of tumors to gefitinib and erlotinib. Proc Natl Acad Sci U S A.

